# Knowledge, beliefs and practices on antibiotic use and resistance among a group of trainee nurses in Sri Lanka

**DOI:** 10.1186/s13104-019-4640-2

**Published:** 2019-09-18

**Authors:** Mathanki Jayaweerasingham, Sacheera Angulmaduwa, Veranja Liyanapathirana

**Affiliations:** 1 Department of Nursing, Faculty of Allied Health Sciences, University of Peradeniya, Peradeniya, Sri Lanka; 2 Department of Microbiology, Faculty of Medicine, University of Peradeniya, Peradeniya, Sri Lanka

**Keywords:** Antibiotic resistance, Nursing students, Sri Lanka

## Abstract

**Objectives:**

Nurses are the main communication link for healthcare messages to hospitalized patients and also play a crucial role in preventing the emergence and spread antibiotic resistant bacteria through antibiotic stewardship and infection control programmes. This requires them to possess correct knowledge and attitudes towards antibiotic use and resistance. This study was carried out to identify the level of knowledge, attitude and practices on antibiotic use and antibiotic resistance among student nurses.

**Results:**

A descriptive cross-sectional study was conducted using a pre-validated, self-administered questionnaire with closed and open ended questions, among 199 student nurses at a government nurses training school in Sri Lanka. Scores and proportions were analysed with non-parametric methods and thematic analysis was done for the qualitative data. The study cohort had a mean knowledge score of 71.9% (SD 14). However, close to 40% believed that taking antibiotics will help to prevent cold from worsening and make recovery faster. Infection control was identified as the main method that nurses can engage in preventing antibiotic resistance. While the knowledge among our study cohort appeared to be good, some misbeliefs were present. Our findings can be used in developing the nursing curricula on antibiotic use and resistance.

## Introduction

Antibiotic resistance is one of the major public health threats faced by the global community. Nurses play a crucial role in combating antimicrobial resistance. They play key roles in antibiotic stewardship and infection control, two main preventive measure of antibiotic resistance [1, 2]. They are one of the main sources of health education to patients and community [3]. Knowledge and necessary attitudes for this role need to be inculcated to nurses during their training period itself. Therefore, identifying the level of knowledge and perceptions among nursing students, and the gaps will enable strengthening of nursing curricula and training programmes. Majority of the nurses working in public hospitals of Sri Lanka are trained by government nursing schools, run by the Ministry of Health while the Faculties of Allied Health Sciences contribute to a lesser proportion of the workforce. Curricula of the nursing schools remain similar across the government nursing schools in Sri Lanka. This study was conducted with the aim of identifying the level of knowledge, beliefs and practices of nursing students at a government nurses training school in Sri Lanka.

## Main text

### Methods

This study was conducted at a Government Nurses’ Training School in the North Western Province of Sri Lanka in 2018. A convenience sampling method was used and all consenting students were recruited for the study. Age, sex, study stream for advanced level examination and the year of study were collected as independent variables.

A pre-validated self-administered questionnaire with sections on knowledge (12 questions), beliefs and practices about the appropriate use of antibiotics, and antibiotic resistance was used. Mean and median scores were calculated and compared using Mann–Whitney U test and Kruskal–Wallis test as the marks were not normally distributed. Percentage giving correct answers were calculated and presented descriptively. For questions related to beliefs and practices, percentages for different categories of answers were calculated. These were compared across the study years using Chi square test. All statistical analysis were performed on SPSS Statistics (v22). A qualitative approach with thematic analysis was conducted to analyze data that were qualitative in nature. All data were manually inspected to identify common themes and proportions expressing similar ideas were calculated.

Total of 199 nursing students were enrolled in this study and among them only 21 (10.5%) were males. All students had studied biological sciences for advanced level examination. Seventy-eight students (39%) were from 1st year, 61 (30.5%) from the 2nd year and 60 (30%) from the 3rd year. All had started their clinical training.

### Results

Hundred and ninety-three students (97%) had heard about antibiotic resistance. Of them, 161 had defined it using their own terms. Their definitions could be categorized into several themes. The commonest them (n = 65, 40.4%) of antibiotic resistance was “bacteria not being responsive or not answering to antibiotic treatment”. Forty (24.8%) participants defined antibiotic resistance as “bacteria resisting the activity of antibiotics”. Seventeen (10.6%) of the participants had defined antibiotic resistance as humans becoming resistant to antibiotics.

Fifteen participants included a qualifier about antibiotic use in their definition of resistance. Nine stated that resistance occurs due to frequent use of antibiotics, five stated that it occurs due to long term use of antibiotics while one had stated that it occurs due to misuse of antibiotics.

A unique finding in our study was that though a high percentage of the participants had heard the term antibiotic resistance qualitative analysis revealed that understanding is not accurate. Some defined antibiotic resistance as humans becoming resistant to antibiotics. Most available studies state that health science students are aware of the term antibiotic resistance [4, 5]. However, most studies have not explored to see the nature of understanding in a qualitative manner. Our findings indicate that qualitative approaches into understanding are more effective in determining the level of awareness. Based on these findings, interventions in curricula can be designed more effectively.

### Knowledge

A total knowledge score percentage was calculated using 12 questions. Marks ranged from 100 to 33.3% with a mean score of 71.9% (SD 14). Considering 72 as the average mark, 115 (57.8%) had above average marks. This compares well with studies done in other countries among health care students, professionals and prescribers [4, 5]. However, “satisfactory” was an interpretation taken by the authors. As antibiotic resistance is of critical importance in the current context, others may have different interpretation on this knowledge score.

The mean and median knowledge scores among females were 71.5% (SD 14.4) and 75. While for males they were 75% (SD 9.8) and 75. Difference between the two sexes was not significant (p = 0.35, Mann–Whitney U test).

The mean knowledge score among the 1st year students was 74.9 (SD 13.5) (median was 75). For the 2nd years, the mean knowledge score was 70.9 (SD 15.8) (median 75). The mean knowledge score among third year students was 69.0 (SD 12) (median 66.7). The difference was statistically significant (p = 0.018, Kruskal–Wallis test). The highest knowledge score was found among first years (74.9%) while the lowest was found among the third years (69%). This interesting finding maybe explained by the longer exposure to actual clinical practice leading to cynicism [6] or the time lapse between the lessons and answering the questionnaire.

The question scoring the lowest percentage of correct answers was on genetic spread of resistance, while the scoring the highest percentage was the one on the activity of antibiotics on bacteria (Table 1). These indicate a need to update the curricula to include the scientific basis for resistance. Without a basic understanding on the scientific context behind resistance, the actual importance of the different interventions may not be apparent to the students leading to lesser adherence to guidelines.

**Table 1:** Correct answer percentages for knowledge on antibiotics and antibiotic resistance

Question	Correct or not	Correct answer (n %)
An antibiotic is active against virus	No	130 (65.3)
An antibiotics is active against bacteria	Yes	182 (91.5)
An antibiotic is active against fungi	No	149 (74.9)
An antibiotic is active against protozoa	No	155 (77.9)
Any antibiotic is active against any type of bacteria	No	144 (72.4)
Antibiotics are indicated for common cold	No	119 (59.8)
Antibiotics are indicated for all types of diarrhoea	No	158 (79.4)
Inappropriate use of antibiotics can lead to antibiotic resistance	Yes	147 (73.9)
Genes responsible for resistance can spread from one bacteria to another	Yes	88 (44.2)
Strengthening infection control measures can reduce the spread of antibiotic resistance	Yes	142 (71.4)
Hand washing can prevent the spread of resistant bacteria	Yes	123 (61.8)
Antibiotics have no side effects	No	180 (90.5)

Among individual questions, 65.3%, 74.4% and 74.9% stated that antibiotics are not active against viruses, fungi and protozoa respectively. While 91.5% stated that antibiotics are active against bacteria, only 72.4% had awareness about the differences in spectrum activity of antibiotics. While these figures compare well with previous studies done among students of health sciences, this is a fundamental point that needs to be corrected if antibiotic misuse for infections are to be prevented [7–9]. The finding that a significant proportion of participants in a range of studies continue to identify that antibiotics are effective against viruses also raises questions on how effective the current teaching methods globally are, in effectively communicating basic factual content.

### Beliefs, attitudes and practices

Seventy-four (37.2%) and 79 (39.7%) believed that taking antibiotics during a cold will prevent it from worsening or help them recover faster (Table 2). There was a significant difference in the proportion of students agreeing to these two statements across the study years.

**Table 2:** Summary of responses—attitude towards antibiotic use and antibiotic resistance

Question	Percentage agreeing				
	Overall (n, %)	1st year	2nd year	3rd year	Significance^a^
When I have a cold I should take antibiotics to prevent it from getting worse	74 (37.2%)	19 (24.4%)	28 (45.9%)	27 (45%)	0.01
When I get any fever, antibiotics help me to get better more quickly	43 (21.6%)	15 (19.2%)	12 (19.7%)	16 (26.7%)	0.52
I believe that antibiotics cure my cold faster	79 (39.7%)	43 (55.1%)	16 (26.2%)	20 (33.3%)	0.001
I would stop taking the prescribed antibiotics if I feel better	64 (72.2%)	20 (25.6%)	24 (39.3%)	20 (33.3%)	0.23
If antibiotics are taken for long time, bacteria become more resistant to antibiotics	177 (88.5%)	72 (92.3%)	54 (88.5%)	51 (85.0%)	0.39
If antibiotics are taken less than the prescribed dose bacteria become less resistant to antibiotics	55 (27.6%)	22 (28.2%)	9 (14.8%)	24 (40.0%)	0.008

^a^Compares difference across all three groups

While in the knowledge section, majority identified misuse of antibiotics lead to emergence of resistance, 72.2% agreed that they may stop taking antibiotics if they start to feel better. Moving on to actual practice, again, close to 50% stated that they may take antibiotics for a shorter duration than indicated. This highlights the gaps in converting knowledge to practice as stated earlier.

### Medication practice on antibiotic use

This section inquired mainly about personal medication use and some practices related to patient care.

In Sri Lankan government health sector, nurses do not play the role of a prescriber yet. Prescription of antibiotics are given by doctors only. However, health care professionals, as well as general public tend to self-medicate [10, 11]. In the study cohort only 116 (58.3%) stated that they always consult a doctor before starting antibiotics.

On being asked the course of action following rapid recovery with an antibiotic, 44 (22.1%) stated that they would stop taking further treatment, 54 (27.1%) stated that they save the remaining antibiotics for another time and 103 (51.8%) stated that they would complete the full course of antibiotics. Furthermore, 124 (62.3%) of the study participants stated that they discard leftover antibiotics while 48 (24.1%) stated that they would give leftover antibiotics to friends. These are practices that need to be actively discouraged, both among health care professionals and the general public.

One of the commonest instances where antibiotic misuse occurs, both over prescription and over the counter medication, is upper respiratory tract infections [12, 13]. Among the study cohort only 59.8% correctly stated that antibiotics are not indicated for common cold (Table 1) while 37.2% agreed that they should take antibiotics during a cold to prevent it worsening (Table 2) and 39.7% agreed that antibiotics cure a cold faster. Interestingly, the highest proportion of students agreeing to the first statement were third years while the highest proportion of students agreeing to the second statement were first years.

We found that antibiotic misuse for upper respiratory tract infections, due to mis-information or false attribution of reasoning is prevalent among the study cohort. Similar findings have been found in other studies too [7]. However, as stated earlier, due to the key role that nurses play as providers of health education, addressing the issue of antibiotic misuse in upper respiratory infections is a timely need.

In relation to patient care related practices, only 78 (39.2%) said they always take culture before patient is given an antibiotic dose and 111 (55.8%) said they always check the expiry date before giving the antibiotic.

### Role of nursing officers

This was assessed by the open-ended question “Write in your own word, in any language that you prefer two things that you as a nursing officer can do to minimize the emergence and spread of resistant organisms”. Hundred and seventy (85.4%) participants replied to this question which had spaces to write 2 answers.

Of the 170, 21 had given two answers while the remaining 149 had given only one answer. The total number of possible actions to be taken identified by participants was 191. These could be categorized into give broad themes. Majority were in infection control (80, 41.9%), antibiotic stewardship (34, 17.8%) and health education (40, 20.9%) while some actions were those that can be taken at a personal level (20, 10.5%) (Table 3).

**Table 3:** Participant identified role of nursing officers in preventing emergence and spread of antibiotic resistance and anticipated barriers

Nurses role in preventing emergence and spread of antibiotic resistance		
Theme	Subtheme	N (%)^a^
Infection control (80, 41.9%)	Practicing hand hygiene	37 (19.4)
	Ensuring environmental hygiene	23 (12.0)
	Barrier nursing	2 (1.0)
	Proper use of sterilization and disinfection methods	12 (6.3)
	Not sharing equipment across patients	4 (2.1)
	Hospital waste management	2 (1.0)
Health education (40, 20.9%)	Educate patients to follow physician advice on antibiotic use	10 (5.2)
	Educate patients on antibiotic course	5 (2.6)
	Educate patients on preventing communicable diseases	5 (2.6)
	Educate patients on personal hygiene and environmental hygiene	3 (1.6)
	Health education—non specified	17 (8.9)
Antibiotic stewardship (34, 17.8%)	Proper administration of antibiotics	14 (7.3)
	Give full course of the antibiotics	6 (3.1)
	Prompting discussion on use of drugs	6 (3.1)
	Ensure quality of drugs	4 (2.1)
	Ensure that patients take the drugs	4 (2.1)
Personal practices (20, 10.5%)	Take antibiotics on prescription only	18 (9.4)
	Personal hygiene	1 (0.5)
	Not sharing antibiotics	1 (0.5)
Universal precautions (17, 8.9%)	Practice of universal precautions	4 (2.1)
	Wearing personal protective equipment	13 (6.8)

Perceived barriers to implement programmes to curb antibiotic resistance		
Theme	N (%)^b^	
Patient behavior related issues	33 (20.2)	
Insufficient human resources/manpower	21 (12.9)	
Insufficient knowledge—patients and staff	19 (11.6)	
Lack of resources and infrastructure (e.g. alcohol hand rub/personal protective equipment)	16 (9.8)	
Hospital waste management issues	14 (8.6)	
None compliance with hand hygiene guidelines	14 (8.6)	
Inability to control antibiotic misuse	13 (8.0)	
Over the counter availability and obtaining of antibiotics	9 (5.5)	
Practices identified are not practical		
Reasons not defined	2 (1.2)	
Due to lack of time	4 (2.4)	
Improper antibiotic administration (injection)	3 (1.8)	
Antibiotic quality related issues	4 (2.4)	
Non adherence to sterilization and disinfection protocols	5 (3.1)	
Not wearing appropriate personal protective equipment	3 (1.8)	
Over-crowding of wards	2 (1.2)	
Other	1 (0.6)	

^a^% Calculated out of the total responses (191)

^b^% Calculated out of total responses (163)

More participants identified infection control or health education related practices as possible contributory role of nursing officers to prevent infection control. However, the percentage of participants identifying antibiotic stewardship related practices were much lower. Antibiotic stewardship is not generally routinely practiced in Sri Lankan hospital set up. This could be a reason why these activities were not identified by the participants. However, antibiotic stewardship is an area where nurses could contribute to prevention of antibiotic resistance [1, 14].

### Anticipated obstacles to implementing activities to minimize emergence and spread of antibiotic resistance

This was assessed by the question “For each of the actions you have mentioned above, write a possible obstacle that you may face in implementing”. There were 163 responses. Most were categorized as patient behavior related issues (33, 20.2%) (Table 3). These included patients not completing antibiotic courses, sharing antibiotics, use of left-over antibiotics, not taking drugs on time. Insufficient man power leading to difficulty in implementing appropriate measures was stated in 21 (11.6%) of the responses while insufficient knowledge on antibiotic use (19, 11.6%) and lack of infrastructure and resources (16, 9.8%) were also cited as possible obstacles to be faced. The commonest reason given by our study cohort was lack of compliance of patients. However, insufficient human resources, infrastructure and knowledge were also identified as possible barriers. These should be taken into consideration in developing institutional programmes for prevention of antibiotic resistance.

## Limitations

Our study was conducted at a single nurses training school in Sri Lanka. However, the curricula remain the same across all the government training schools. We did not inquire the source for the information they received, any formal training or look at the nursing school curricula. The term antibiotic though contextually used to denote antibacterials, maybe literally interpreted to include anti-fungal and anti-protozoal drugs too. This may have led to some misinterpretation of the questions on this section.

However, the main strength in our study is that we included a qualitative component, that helps to explore the ideas prevalent among the study population, that would not have been revealed in a quantitative study.

## Data Availability

The datasets used and analysed during the current study are available from the corresponding author on reasonable request.

## Abbreviation

SD: standard deviation
